# Assessing the sensitivity and predictive value of wastewater in detection of Hepatitis A cases in San Diego County

**DOI:** 10.1371/journal.pone.0342229

**Published:** 2026-02-18

**Authors:** Aishwarya Ramesh, Ravi Goyal, Sarah Stous, Hannah R. Thomas, Seema Shah, Eliah Aronoff-Spencer, Mark E. Beatty, Natasha K. Martin

**Affiliations:** 1 Division of Infectious Diseases and Global Public Health, University of California San Diego, La Jolla, California, United States of America; 2 County of San Diego Health and Human Services Agency, San Diego, California, United States of America; 3 Design Lab, University of California San Diego, La Jolla, California, United States of America; 4 Population Health Sciences, University of Bristol, Bristol, United Kingdom; Yamagata University Faculty of Medicine: Yamagata Daigaku Igakubu Daigakuin Igakukei Kenkyuka, JAPAN

## Abstract

Hepatitis A virus (HAV) remains a significant public health concern in the United States. Because infected individuals shed virus through stool, HAV can be detected in wastewater. Shedding occurs prior to the onset of symptoms that lead to clinical diagnosis, highlighting the potential of wastewater as an early case detection tool. This analysis aims to quantify key diagnostic metrics of wastewater surveillance for detecting HAV cases, which have not been previously defined. Utilizing wastewater data from the Point Loma Wastewater Treatment Facility in San Diego County, which serves around 2.2 million people, we assessed the sensitivity, positive predictive value (PPV), and negative predictive value (NPV) of wastewater HAV signals (positive/negative) in identifying shedding cases over a 308-day period. The number of people shedding virus on a given day was estimated through confirmed cases and presumed shedding intervals (2 weeks before and 1 week after symptom onset) and compared to wastewater signals. The sensitivity in detecting at least one shedding case on a given day using observed wastewater signals was 48.1%. Reclassifying the wastewater signal using simple data aggregations yielded sensitivities from 67.3% to 84.6%. Sensitivity increased as more individuals were shedding virus. The highest PPV (52.2%) and NPV (74.2%) were observed when a 5-sample trimmed centered average was used to reclassify the wastewater signal, indicating the utility of this preprocessing method. Conditional on clinical case detection and shedding assumptions, our study demonstrates that wastewater is a promising tool, providing signals that can inform public health surveillance.

## Introduction

Hepatitis A virus (HAV) remains a significant public health concern in the United States. Although foodborne outbreaks continue to occur, direct person-to-person contact among people who use drugs (PWUD), persons experiencing homelessness (PEH), and men who have sex with men (MSM) are the leading sources of cases [1]. Though a vaccine is available, it was only added to the childhood immunization schedule in 2006, therefore most adults in the US over the age of 19 have not been vaccinated [1]. Due the predilection for Hepatitis A virus (HAV) to infect the liver, fecal-oral transmission is the predominant route of transmission [1]. Consequently, HAV virus can be detected in wastewater; such surveillance data is reported for multiple U.S. jurisdictions on the WastewaterSCAN Dashboard [2,3]. However, key diagnostic metrics of wastewater surveillance for detecting HAV cases are unknown. This analysis examines the utility of wastewater in detecting HAV cases in San Diego County.

Wastewater-based epidemiology (WBE) for surveillance of HAV has previously been used as evidence of the extent to which HAV is circulating within a region and its water sources [4–6]. High prevalence in wastewater is often considered an indicator of HAV endemicity [6]. HAV wastewater samples have also been genotyped to assess which specific subtypes and strains are circulating within a community. These sequences are often compared to sequences from patients to confirm conclusions from wastewater sampling [7–9]. For instance, sequencing of wastewater samples from a plant in Sweden during the 2013 HAV outbreak in Scandinavia yielded two strains of the subtype IB similar to strains causing the broader Scandinavian outbreak. This indicated ongoing transmission of these regional outbreak strains within the local wastewater catchment area, which was later confirmed by the presentation of clinical cases with the outbreak strain [7].

Increases in wastewater HAV nucleic acid concentrations have been found to precede clinical cases temporally [7,10–13]. However, this lead-time is influenced by many factors, including public health and sanitation infrastructure, and therefore may vary by geographic area. For example, in the 2013 Swedish outbreak, HAV strains were found in wastewater 3–12 weeks before patients with similar strains were clinically diagnosed [7]. Wastewater monitoring of the city of Cordoba, Argentina revealed that increases in circulating wastewater HAV preceded clinical cases by up to 2 months in 2017 and 2018, and up to 6 weeks in 2022 [10]. In North American settings, the time between detecting HAV in wastewater and clinically diagnoses tends to be somewhat shorter. A study conducted during the 2017 HAV outbreak in Detroit, Michigan—which resulted in 920 cases as of December 2019—showed that wastewater increases were correlated to cases 7 days later (Spearman correlation of 0.55) [11]. A study in Canada corroborated this shorter period, finding a statistically significant positive correlation between cases of HAV and HAV RNA concentration in wastewater one week prior at the Guelph Water Resource Recovery Center, though the number of cases was small (n = 4) [12]. In addition, a case study (n = 20) in Maine found—through a cross-correlation analysis—that average wastewater concentration leads clinical cases by one week [13]. Finally, a recent study in Los Angeles found that HAV wastewater measurements increase concurrently with increases in cases (n = 11) [14].

Increases of HAV in wastewater are not always correlated with increases in clinical cases, nor are strains found in wastewater always consistent with strains found in clinical settings. For example, during an outbreak in Italy in 2012 and 2013 surveillance of 19 wastewater treatment plants (WWTPs) detected positive HAV signals in two WWTPs that did not correspond to any known cases. There were also 58 cases within wastewater catchments that had no corresponding wastewater signal. Moreover, strains found in wastewater and in clinical cases did not match, with HAV genotype IA dominating clinically (90.2% of clinical samples) but genotype IB overrepresented in wastewater (81.8% of wastewater samples) [9]. During the 2013 HAV outbreak in Sweden, an IB strain similar to those from the Middle East was identified in wastewater, but not in clinical cases, while an IA strain was identified in a patient but not in wastewater samples [7]. Finally, during an outbreak at a Dutch primary school, genotype IA was detected in wastewater but was not associated with any cases [15]. While differences in viral RNA concentration and extraction methods can affect which strains are identified, this discrepancy between patient strains and wastewater observations also indicates that the population contributing to wastewater signals and those who ultimately present for care are not necessarily concordant. This highlights the need to understand the relationship between clinical cases of HAV and wastewater signals.

A recent national study in the United States of 191 WWTPs in 40 states and the District of Columbia aimed to characterize the presence of HAV in wastewater, and its connection to clinical cases from the National Notifiable Disease Surveillance System. The study found that weekly wastewater HAV nucleic acid concentrations and weekly positivity rates had modest correlations (Kendall’s tau of 0.20 and 0.33 respectively, P < 0.05) with case data [13]. These study results demonstrate that WBE can provide public health departments insight on concurrent disease burden within the community. However, the performance characteristics of wastewater (namely the sensitivity, positive predictive value and negative predictive value) in identifying cases within the population need to be quantified.

San Diego County experienced a large outbreak of HAV in 2016–2018, with 592 cases and 20 deaths, the majority of which were PEH and/or PWUD [16]. In 2023, the County experienced elevated cases of HAV (n = 45) primarily among PEH (46.7% of cases). Given the scale of past outbreaks, timely detection of HAV cases is a significant public health priority in San Diego County. Wastewater HAV signals can act as an early warning system, enabling the detection of HAV cases as and when they begin shedding rather than after an outbreak-level situation has occurred. Thus, this analysis aimed to ascertain the sensitivity and predictive value of wastewater HAV signals in detecting individuals who are presumed shedding HAV, in a setting where significant outbreaks have occurred.

## Materials and methods

### Data sources

Wastewater data were obtained from the WastewaterSCAN Dashboard for the San Diego Point Loma Wastewater Treatment Facility between September 10, 2023, and July 13, 2024 [2,3]. The Point Loma Wastewater Treatment Facility treats more than 662,000 kiloliters of water in a day, in a catchment area that covers around 1,170 square kilometers and serves more than 2.2 million people [17]. For each day with wastewater sampling (samples collected Sunday, Monday, and Wednesday), the WastewaterSCAN Dashboard presents two wastewater values: 1) the Pepper Mild Mottle Virus (PMMoV) normalized HAV nucleic acid level and 2) a 5-sample trimmed centered average of the PPMoV normalized HAV nucleic acid levels (calculated by taking five consecutive samples centered on a day, dropping the minimum and maximum and averaging the rest).

**Fig 1:**
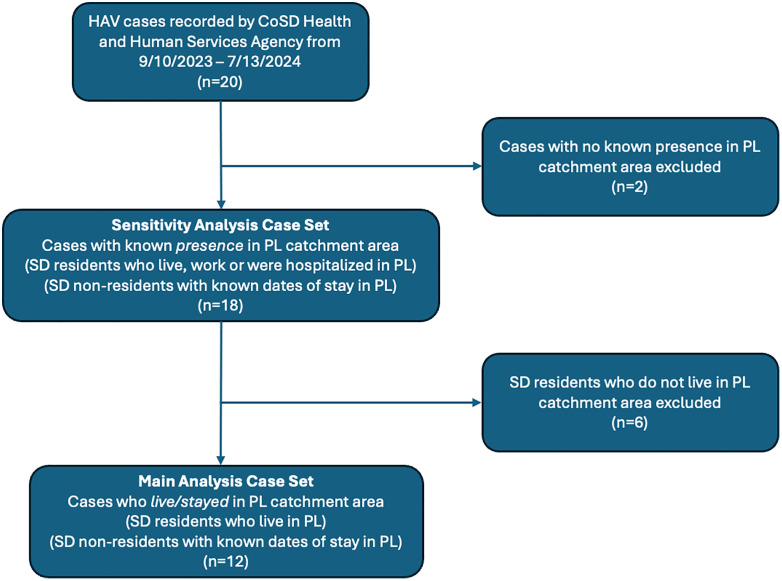
Flow Chart of Case Inclusion. The main analysis includes confirmed cases who were present either through residence or known dates of stay in the PL catchment area during their period of shedding (n = 12). This includes SD residents who reside within the PL area, and SD non-residents who were staying in PL during their presumed shedding period. The sensitivity analysis includes all cases with known presence in the PL catchment area during their period of presumed shedding (n = 18). In addition to the cases from the main analysis, this includes SD residents who worked or were hospitalized PL (n = 6) who were excluded from the main analysis as they did not reside in PL. Cases with no known presence in the PL catchment during their presumed shedding period were excluded regardless of SD county resident status (n = 2).

We obtained information on confirmed HAV cases in San Diego County during the period of wastewater surveillance. Case data were accessed beginning on November 13^th^, 2024, and all data were de-identified prior to acquisition for analysis. According to CDC’s 2019 case definition, a confirmed HAV case must fulfill one of the following sets of criteria: 1) clinical criteria for HAV—defined as acute illness with a discrete onset of HAV symptoms (i.e., fever, headache, malaise, anorexia, nausea, vomiting, diarrhea, abdominal pain, or dark urine), and either jaundice or elevated bilirubin levels (≥3.0 mg/dL) or elevated serum alanine aminotransferase (ALT) levels (>200 IU/L), and the absence of a more likely diagnosis—as well as positive immunoglobulin M (IgM) antibody to HAV (anti-HAV); 2) HAV RNA detected by a nucleic acid amplification test (NAAT) such as PCR, or 3) clinical criteria and occurs in a person who has had direct contact with a laboratory confirmed HAV case 15–50 days prior to symptom onset [18]. For each case, we received information on its episode date, which was defined as the earliest date of: symptom onset, specimen collection, diagnosis, death, or report received. Cases were classified based on residence: San Diego resident residing within the Point Loma (PL) catchment area, San Diego resident not residing within the PL catchment area, nonresident with known dates of stay within the PL catchment area, and nonresident with no known dates of stay within the PL catchment area. For cases who were San Diego County residents, but not PL residents, we were also provided information regarding epidemiologic linkage to the PL catchment area such as employment or hospitalization. Such information was based on County of San Diego (CoSD) HAV case ascertainment data. For the main analysis, we only include individuals who were San Diego County residents residing within the PL catchment area or who were nonresidents with known dates of stay within the PL catchment area. As a sensitivity analysis, we included San Diego County residents outside of the PL catchment area with known dates of presence in the area. Any cases with no known presence in the PL catchment area were excluded from all analyses. A flow chart showing HAV case inclusion criteria for this analysis is shown in Fig 1 below.

### Data analysis

The primary analysis was a comparison of binary wastewater signals (HAV positive or negative) and the presence of an individual presumed to be shedding virus on a given day. Below we provide details on both quantities.

#### Methods of calculating binary wastewater signal.

There are several considerations when assessing Hep A disease burden in San Diego County using routinely sampled wastewater surveillance data. First, samples are often “grab” samples taken at one point in time, which may not reflect all individuals in the catchment [3]. Second, the transit time of sewage from source to the Point Loma facility ranges on the order of minutes/hours (i.e., same day detection) up to 3 days for those at the furthest distance (50 miles, with the lowest design flow velocity of 1 ft per second). Due to these temporal factors, we examine multi-sample average classifications of wastewater HAV positivity, in addition to same-day detection. Specifically, we classified each day where wastewater was sampled (testing day) as either HAV positive or negative using nine different classification methods and assessed the performance of these metrics. Below we list each method and how it classified each day when wastewater was sampled as wastewater HAV positive or negative.

- **Method 1 – Observed:** The testing day was classified as wastewater HAV positive if the sample had a nonzero HAV nucleic acid level.
- **Method 2 – Weekly classification:** The testing day was classified as positive if any sample that week was positive.
- **Method 3–5-sample trimmed centered average:** The testing day was classified as positive if the 5-sample trimmed centered average was nonzero. The 5-sample trimmed centered average is reported directly on the WastewaterSCAN Dashboard and is calculated by taking 5 consecutive samples centered on a day, dropping the minimum and maximum and averaging the remainder.
- **Methods 4–6–3, 5, or 7-sample centered average:** The testing day was classified as positive if the 3, 5, or 7-sample centered average was nonzero. At boundaries, available values were averaged centered on the day in question.
- **Methods 7–9–3, 5, or 7-sample running average:** The testing day was classified as positive if 3, 5, or 7 consecutive samples ending on a testing day were nonzero. At boundaries, available values were averaged ending on the day in question.

#### Method of estimating number of individuals shedding on a given day.

The number of individuals shedding virus was estimated for each day using CoSD HAV surveillance data. Centers for Disease Control and Prevention (CDC) reported that individuals typically shed virus through stool from 2 weeks before to 1 week after onset of symptoms [1]. We used the episode date reported by CoSD as a proxy for symptom onset. For non-resident cases identified in the CoSD data, we truncated the shedding period to reflect known dates of presence in the PL catchment area. For the primary analysis, we dichotomized the number of individuals shedding as either none or at least one person.

#### Primary analyses.

We performed the following analysis for each of the nine methods of calculating binary wastewater signal. For each sampled day, the binary wastewater classification (HAV + / HAV-) and the presence of an individual shedding were compared to classify the day as a true positive (TP), false negative (FN), true negative (TN), and false positive (FP). Specifically, true positives were days with a HAV positive wastewater signal and one or more individuals shedding, false negatives were days with a negative wastewater signal but one or more individuals shedding, true negatives were days with a negative wastewater signal and no individuals shedding, and false positives were days with a positive wastewater signal but no individuals shedding [19].

Given the total number of TP, FN, TN and FP, the sensitivity, positive predictive value (PPV) and negative predictive value (NPV) of wastewater signals in identifying one or more individuals shedding were calculated. Sensitivity is the probability of a wastewater signal given one or more individuals shedding (TP/ (TP + FN)). PPV is the probability that one or more individuals were shedding given a positive wastewater detection (TP/ (TP + FP)), while NPV is the probability that no individuals were shedding given the absence of a wastewater signal (TN/ (TN + FN)) [19] A 95% CI was calculated for each metric using a normal approximation for all 9 wastewater classification methods.

#### Sensitivity analyses.

As our calculated performance metrics are necessarily conditional on shedding assumptions, assay limits of detection and case attribution to the PL catchment, multiple sensitivity analyses were conducted. Firstly, we varied the threshold for the number of individuals shedding (≥2, ≥ 3 or ≥4 individuals); the primary analysis investigated detecting one or more at baseline. We also conducted analysis changing the duration of the shedding period; the primary analysis set this as 2 weeks before to 1 week after onset of symptoms. Prior studies have found variation in shedding period from 2.5 weeks before and up to 11 weeks after symptom onset, typically tapering off between 3.5–5 weeks after symptom onset. Maximal viral shedding has been known to occur around the time of symptom onset [20,21]. When varying the shedding period, we aimed to capture both the narrow period of maximal shedding around symptom onset, as well as individuals who may shed for much longer. As a result, we truncated the shedding prior to symptom onset from 2 weeks to 1 week to represent a potential shorter period of high shedding. Specifying alternative shedding periods after symptom onset was more complex, due to the large range reported. We decided to investigate an extended period of 4 weeks after symptom onset, in line with evidence that shedding stops between 3.5–5 weeks after symptom onset [20,21]. Therefore, in addition to the primary analysis, we assess the following shedding periods: 1 week before and after, 1 week before and 4 weeks after, and 2 weeks before and 4 weeks after symptom onset. An additional sensitivity analysis was conducted on the limits of detection threshold. The lower limit of detection used to classify results in our main analysis is 1,000 copies HAV/g [13]. To account for background noise and measurement variation, we classified any wastewater samples with HAV concentrations below 5,000 cp/g, 7,500 cp/g and 10,000 cp/g respectively as negative. A final sensitivity analysis was conducted including San Diego County cases who did not reside in the PL catchment area, but who worked, were hospitalized or were present in PL during their presumed shedding period according to county epidemiological data. For these cases, only dates they were known to be present in PL within their shedding period were classified as shedding days. Python (version 3.11.5; Python Software Foundation) was used for all analyses. The datasets and code used in the main analysis of this study are publicly available at https://github.com/a2ramesh/Wastewater_Utility_HAV_SDC.

## Results

**Fig 2:**
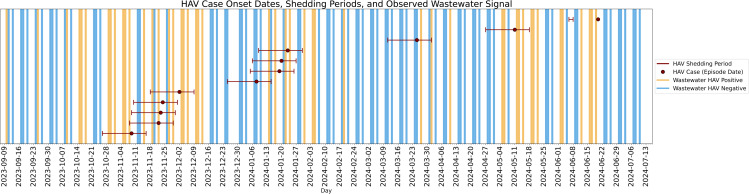
Observed Wastewater Signal, Cases and their Shedding Period. In the 308 days from September 10^th^ 2023 to July 13^th^ 2024 used in this analysis, there were 12 cases of HAV reported by San Diego County, represented above by points on their respective episode dates. In that same period, wastewater was sampled from the Point Loma Wastewater Treatment Facility a total of 131 times, with positive sample days being shaded orange and negative sample days in blue. A total of 48 samples tested positive for HAV nucleic acids. Each case had a 3-week long shedding period which intersected with the shedding periods of other cases, represented by the horizontal bars on each case.

**Fig 3:**
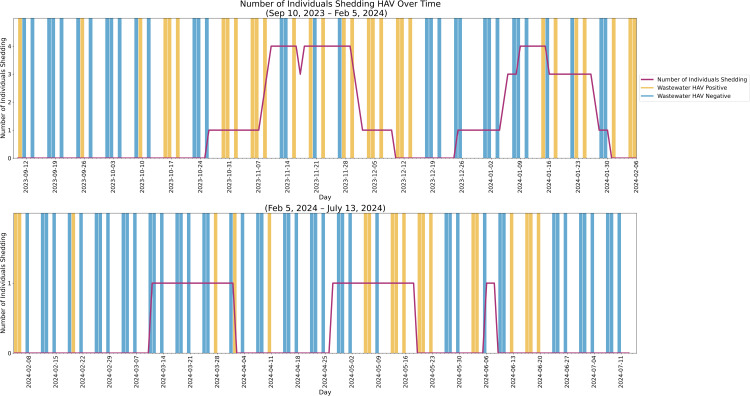
Distribution of Shedding and Observed Wastewater Signal. The number of individuals shedding is shown in purple overlaid on the Observed wastewater signal during the study period. Positive sample days are shaded orange and negative sample days are blue. The number of individuals shedding at any given time ranged from 0 to 4 individuals.

In the 308 days analyzed, wastewater was sampled 131 times of which 48 samples (36.6%) had nonzero HAV nucleic acid levels (Fig 2). During that period, 12 cases of Hep A were reported in San Diego County associated with the Point Loma Wastewater Sampler catchment area. These cases consisted of 10 people residing in the Point Loma Wastewater Sampler catchment area, and 2 San Diego County non-residents staying in the catchment area. Of the 12 cases, one non-resident case was a PEH. The estimated shedding period for each of the 12 cases, as well as wastewater signals in the same period, are shown in Fig 2. There were a total of 129 days (41.9% of study duration) with at least one individual shedding. The overlapping shedding periods resulted in a minimum estimate of 1 to a maximum estimate of 4 individuals shedding during the measured period. The distribution of individuals shedding over time is shown below in Fig 3.

An additional 6 cases of Hep A who did not reside in the Point Loma catchment area but had known affiliations to the area (S1 Fig) and resided in San Diego County were reported during this period. Of these 18 total cases, 16 (88.9%) had an episode date that corresponded to the date of symptom onset. The 2 cases that had a different episode date were included in the main analysis and had episode dates corresponding to date of lab specimen collection (19 Jan 2024) and date of received report of a case (20 Jun 2024) respectively.

### Observed wastewater signal sensitivity, PPV and NPV (Method 1)

When testing days were classified as HAV positive based on the observed wastewater measurement from that day (Method 1), the sensitivity of wastewater in detecting at least one case was 48.1% (95% CI: 34.5%–61.7%), the PPV was 52.1% (95% CI: 38.0%–66.2%), and the NPV was 67.5% (95% CI: 57.4%–77.5%).

### Additional wastewater signal classifications (Methods 2–9)

Alternative classification of the wastewater signal (Methods 2–9) improved sensitivity compared to the observed wastewater measurement (Method 1). The PPV for Methods 2–9 were generally lower and NPV was mixed compared to the observed wastewater signal. The 5-sample trimmed centered average yielded the highest PPV of 52.2% (95% CI: 40.4%–64.0%) and NPV of 74.2% (95% CI: 63.3%–85.1%) while retaining a sensitivity of 69.2% (95% CI: 56.7%–81.8%). The highest sensitivity of the 9 classification methods was achieved using the 7-sample centered average (84.6%; 95% CI: 74.8%–94.4%) but the PPV (40.7%; 95% CI: 31.5%–50.0%) and NPV (65.2%; 95% CI: 45.8%–84.7%) declined significantly. Methods averaging more samples increased sensitivity, but decreased PPV and NPV. Centered averages performed better than rolling averages for the same period. Sensitivity, PPV and NPV using all wastewater classification methods are shown in Table 1.

**Table 1:** Sensitivity, Positive Predictive Value (PPV) and Negative Predictive Value (NPV) for Detecting At Least One Case for All Wastewater Classification Methods.

Wastewater Signal Classification Strategy	Sensitivity % (95% CI)	PPV % (95% CI)	NPV (% (95% CI)
Observed wastewater signal	48.1 (34.5, 61.7)	52.1 (38.0, 66.2)	67.5 (57.4, 77.5)
Wastewater signal reclassified if any positive in the week	71.2 (58.8, 83.5)	49.3 (38.0, 60.6)	73.2 (61.6, 84.8)
Wastewater signal reclassified based on 5-sample trimmed centered average (default)	69.2 (56.7, 81.8)	52.2 (40.4, 64.0)	74.2 (63.3, 85.1)
Wastewater signal reclassified based on 3-sample centered average	73.1 (61.0, 85.1)	47.5 (36.6, 58.4)	72.5 (60.3, 84.8)
Wastewater signal reclassified based on 5-sample centered average	78.8 (67.7, 89.9)	42.3 (32.4, 52.1)	67.6 (51.9, 83.4)
Wastewater signal reclassified based on 7-sample centered average	84.6 (74.8, 94.4)	40.7 (31.5, 50.0)	65.2 (45.8, 84.7)
Wastewater signal reclassified based on 3-sample rolling average	67.3 (54.6, 80.1)	43.2 (32.4, 54.0)	66 (52.9, 79.1)
Wastewater signal reclassified based on 5-sample rolling average	67.3 (54.6, 80.1)	35.4 (25.9, 44.8)	46.9 (29.6, 64.2)
Wastewater signal reclassified based on 7-sample rolling average	71.2 (58.8, 83.5)	33.3 (24.6, 42.1)	25 (6.0, 44.0)

### Sensitivity analyses

**Fig 4:**
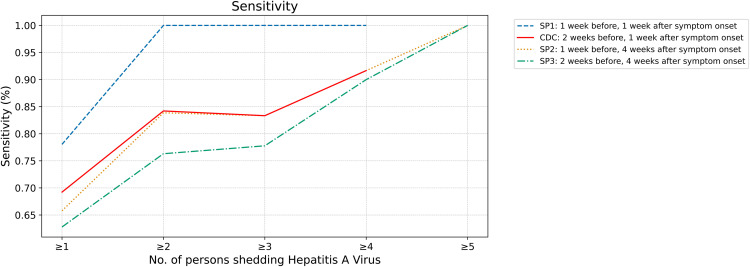
Sensitivity Calculated Using Wastewater signal reclassified based on 5-sample trimmed centered average. Sensitivity increases as the number of individuals shedding increases across shedding periods (Blue dashed line: 1 week before and 1 week after symptom onset; Red solid line: 2 weeks before and 1 week after symptom onset; Orange dotted line: 1 week before and 4 weeks after symptom onset; Green dash-dotted line: 2 weeks before and 4 weeks after symptom onset). Sensitivity for identification of at least one case is higher for shorter shedding periods.

When varying shedding period length, shorter durations had higher sensitivities due to a lower number of false negatives. The sensitivity also increased with increasing number of individuals shedding. These trends are illustrated in Fig 4.

The increase in sensitivity as the number of individuals shedding increased was consistent across classification methods as well, with all methods except Method 1 (Observed wastewater signal) having a sensitivity greater than 90% with 4 or more shedding cases (S1 Table). The results of the sensitivity analysis varying the lower threshold of detection (using Method 1, the Observed wastewater signal) are shown in S2 Table. As the threshold for a positive wastewater signal increases, the sensitivity decreases, while the PPV increases, and the overall number of negatives (including TN and FN) increase while the total number of positives (TP and FP) decrease. The improvement in PPV indicates a higher probability of a true shedding case given a positive wastewater signal when higher thresholds are used. Finally, considering additional cases not residing in the PL area along with the 5-sample trimmed centered average increased the PPV (59.4%) but reduced sensitivity (64.1%) and NPV (62.9%) compared to the 5-sample trimmed centered average in the primary analysis. This demonstrates that additional cases are responsible for some wastewater signal, but not all their shedding may have been in PL (S1 Fig).

## Discussion

We found that wastewater surveillance can be a useful tool for HAV case detection, and minimal data preprocessing can increase sensitivity considerably. A default classification available on the WastewaterSCAN dashboard is the 5-sample trimmed centered average, which our analysis shows has a sensitivity of 69% for detecting at least one case and maintains a PPV of 52% and NPV of 74%.

Our study supports other studies highlighting the utility of wastewater surveillance for HAV cases in the United States. The study examining an outbreak of HAV in Los Angeles County beginning in March 2024 found disproportionate (3x and 7x higher) HAV nucleic acid concentrations in two wastewater plants when 7 cases were reported, compared to other months where 2–5 cases were reported [14]. Studies in Detroit in 2017, and nationally, also found positive correlations between wastewater signals and clinical cases (Spearman correlation of 0.55 and Kendall’s tau of 0.33 respectively) [11,13]. Although these studies find positive associations between wastewater nucleic acid levels and cases, they did not specifically examine characteristics such as sensitivity, PPV, and NPV.

Strengths of our study include that we were able to precisely classify cases into the catchment area of the wastewater sampler and compare sensitivity, PPV and NPV in a quantitatively robust manner. The multiple sensitivity analyses conducted illustrate changes in results if any given assumption (ex. shedding period length, case attribution to catchment, assay limits of detection) failed. Overall, these analyses can provide a framework for systematically quantifying the performance of a wastewater treatment plant in detecting community disease burden.

The sensitivity of wastewater in detecting at least one HAV case shedding virus (48.1%) in our study is higher than recent reports of wastewater for Mpox detection (32% for detecting one or more individuals shedding the virus using data from CDC’s National Wastewater Surveillance System) [19]. Our analysis shows comparatively improved performance, although increased case ascertainment for Mpox due to rash presentation and differential wastewater infrastructure must be considered. When reclassifying the signal, our findings are similar to a study on poliovirus detection in wastewater in Pakistan (sensitivity of around 60%) [22]. There is also evidence in our study that wastewater may be able to detect a single shedding case of HAV. Multiple past studies of wastewater sensitivity have been focused on SARS-CoV-2 in the community, and typically a threshold number of cases were needed before wastewater detection occurred. For instance, one study identifying COVID-19 cases through wastewater signals in Vancouver Island, Canada found a sensitivity of 75%, but only when there were above 17−19 cases per 100,000 people per week [23]. Another study in New Zealand found that the probability of SARS-CoV-2 RNA detection in wastewater was 28% and 41% when case rates were 5 and 10 infectious cases per 100,000 population respectively [24]. It seems that HAV can be identified more easily, potentially because it is a non-enveloped enterically shed virus and is more stable in the environment than enveloped viruses like SARS-CoV-2 [25].

However, a notable limitation of this study is the potential for case under-ascertainment due to asymptomatic cases, individuals not seeking care or misdiagnoses by clinical staff. Up to 30% of adult HAV cases are asymptomatic according to the CDC [1], and the disease occurs primarily in populations like PEH that have structural barriers to accessing care, such as negative prior experiences with healthcare or competing basic needs [26]. Therefore, signals that appear to be coming from a single shedding case may be the result of multiple concurrently shedding cases, only one of which was clinically detected. This inflates our ability to detect a single case in this study. Moreover, missed cases (asymptomatic, undiagnosed or non-recorded) may also be responsible for higher FP (days with a positive wastewater signal but no known shedding cases), which would deflate PPV and sensitivity. The stability of HAV in the environment [25] might also be a source of FP, as HAV is able to persist in water for up to 90 days at a wide range of temperatures [27]. This would result in a positive wastewater signal long after shedding has occurred.

Another major limitation is the potential for misclassification of shedding days due to individual variation in shedding periods and stool patterns, equating of episode date with date of symptom onset, and potential travel of individuals into and out of the catchment area. Shedding misclassification (including the two cases whose episode dates were date of specimen collection or date of case report) would presumably inflate FN and deflate TP, leading to a lower sensitivity and NPV. This is because a shifted shedding period results in areas where we presume shedding, but where no signals are observed (but could have been observed if the shedding period was correctly identified). As previously mentioned, the reported sensitivity, PPV and NPV must be interpreted conditional on these assumptions.

While reclassification of wastewater signals improved our sensitivity, all reclassification methods were not equal. Thus, the choice of reclassification method depends on the goals of a health jurisdiction. If complete case identification regardless of false positives is a priority, sensitive methods averaging more samples may be preferred. Similarly, rolling averages may be preferred for quick action, as they do not use future data. Regardless of the classification method used, sensitivity increased with the number of individuals shedding, indicating that a large outbreak would be highly likely to be detected in the wastewater.

While this study primarily focused on concurrent detection of cases, future studies can examine whether wastewater can robustly be used as a leading (or lagging) indicator for HAV outbreaks, as the significant advantage wastewater surveillance provides is the detection of a cluster prior to an outbreak-level situation. Furthermore, this study assumed that individuals shed virus uniformly over the course of their shedding period. Future studies may repeat these analyses with more realistic shedding distributions to better understand the relationship between clinical cases and wastewater signals.

## Conclusions

Wastewater is a promising tool for HAV surveillance, which demonstrates variation in sensitivity with different data aggregation methods. Utilization of wastewater surveillance for the detection of HAV within a community can inform public health practice in case finding and outbreak investigations.

## Supporting information

S1 Fig HAV Cases and Association to Point Loma Catchment. A total of 6 cases were added that worked, were hospitalized or were present during their presumed period of illness. The episode dates and shedding periods of these cases, along with the observed wastewater signal are shown. When these cases are included, there is a higher number of true positives and false negatives. This indicates that the additional cases could be responsible for some wastewater signals, but not all their shedding potentially occurs in the PL catchment area.
(PDF)

S1 Table Sensitivity in Percent as Number of Individuals Shedding Increases for All Wastewater Classification Methods. (DOCX)

S2 Table Sensitivity in Percent at Varying Detection Thresholds using Observed Wastewater Signal. (DOCX)

## References

[1] Foster MA, Haber P, Nelson NP. Chapter 9: Hepatitis A. Epidemiology and Prevention of Vaccine-Preventable Diseases (The Pink Book). 14th ed. Atlanta: Centers for Disease Control and Prevention; 2021 [cited 2024 Mar 11]. Available from: https://www.cdc.gov/pinkbook/hcp/table-of-contents/chapter-9-hepatitis-a.html

[2] Boehm AB, Wolfe MK, Wigginton KR, Bidwell A, White BJ, Hughes B, et al. Human viral nucleic acids concentrations in wastewater solids from Central and Coastal California USA. Sci Data. 2023;10(1):396. doi: 10.1038/s41597-023-02297-7 37349355 PMC10287720

[3] WastewaterSCAN. Stanford University Sewer Coronavirus Alert Network (SCAN). https://www.wastewaterscan.org/en. 2024.

[4] Girón-Guzmán I, Cuevas-Ferrando E, Barranquero R, Díaz-Reolid A, Puchades-Colera P, Falcó I, et al. Urban wastewater-based epidemiology for multi-viral pathogen surveillance in the Valencian region, Spain. Water Res. 2024;255:121463. doi: 10.1016/j.watres.2024.121463 38537489

[5] Raya S, Tandukar S, Kattel HP, Sharma S, Sangsanont J, Sirikanchana K, et al. Prevalence of hepatitis A and E viruses in wastewater in Asian countries. Sci Total Environ. 2024;951:175473. doi: 10.1016/j.scitotenv.2024.175473 39142413

[6] Toancha K, Borges A, Lázaro L, Teixeira N, Lima AK, Gonçalves A, et al. Wastewater-based surveillance for Hepatitis A virus, Enterovirus, Poliovirus, and SARS-CoV-2 in São Tomé and Príncipe: A pilot study. Sci Total Environ. 2024;955:176923. doi: 10.1016/j.scitotenv.2024.176923 39427898

[7] Hellmér M, Paxéus N, Magnius L, Enache L, Arnholm B, Johansson A, et al. Detection of pathogenic viruses in sewage provided early warnings of hepatitis A virus and norovirus outbreaks. Appl Environ Microbiol. 2014;80(21):6771–81. doi: 10.1128/AEM.01981-14 25172863 PMC4249052

[8] Bisseux M, Colombet J, Mirand A, Roque-Afonso A-M, Abravanel F, Izopet J, et al. Monitoring human enteric viruses in wastewater and relevance to infections encountered in the clinical setting: a one-year experiment in central France, 2014 to 2015. Euro Surveill. 2018;23(7):17–00237. doi: 10.2807/1560-7917.ES.2018.23.7.17-00237 29471623 PMC5824128

[9] La Rosa G, Libera SD, Iaconelli M, Ciccaglione AR, Bruni R, Taffon S, et al. Surveillance of hepatitis A virus in urban sewages and comparison with cases notified in the course of an outbreak, Italy 2013. BMC Infect Dis. 2014;14:419. doi: 10.1186/1471-2334-14-419 25074676 PMC4122772

[10] Fantilli A, Cola GD, Castro G, Sicilia P, Cachi AM, de Los Ángeles Marinzalda M, et al. Hepatitis A virus monitoring in wastewater: A complementary tool to clinical surveillance. Water Res. 2023;241:120102. doi: 10.1016/j.watres.2023.120102 37262946

[11] McCall C, Wu H, O’Brien E, Xagoraraki I. Assessment of enteric viruses during a hepatitis outbreak in Detroit MI using wastewater surveillance and metagenomic analysis. J Appl Microbiol. 2021;131(3):1539–54. doi: 10.1111/jam.15027 33550682

[12] Chettleburgh C, McDougall H, Parreira V, Goodridge L, Habash M. Seasonality of enteric viruses and correlation of hepatitis a virus in wastewater with clinical cases. Sci Total Environ. 2025;967:178862. doi: 10.1016/j.scitotenv.2025.178862 39955939

[13] Zulli A, Wolfe MK, Boehm AB, Duong D, Hughes B, Bakker KM, et al. Detection of Hepatovirus A (HAV) in wastewater indicates widespread national distribution and association with socioeconomic indicators of vulnerability. Environ Sci Technol. 2024;58(10):4123–34. doi: 10.1021/acs.est.3c08974 PMC11580403 39475316

[14] Braunfeld JB, Dao BL, Buendia J, Amiling R, LeBlanc C, Jewell MP, et al. Notes from the Field: Genomic and wastewater surveillance data to guide a hepatitis A Outbreak Response - Los Angeles County, March 2024-June 2024. MMWR Morb Mortal Wkly Rep. 2025;74(5):66–8. doi: 10.15585/mmwr.mm7405a3 39977374 PMC12370257

[15] de Jong M, van der Loeff MFS, Schilperoort R, Vennema H, van der Weijden C, Langeveld J, et al. Use of passive samplers as sewage surveillance tool to monitor a hepatitis A outbreak at a school in Amsterdam, the Netherlands, Oct 2022 - March 2023. BMC Infect Dis. 2024;24(1):1044. doi: 10.1186/s12879-024-09938-1 39333937 PMC11430438

[16] County of San Diego. Hepatitis A outbreak. https://www.sandiegocounty.gov/content/sdc/hhsa/programs/phs/community_epidemiology/dc/Hepatitis_A/outbreak.html

[17] City of San Diego. Point Loma Wastewater Treatment Plant. https://www.sandiego.gov/public-utilities/water-quality/water-wastewater-facilities/point-loma. Accessed 2025 July 13.

[18] Centers for Disease Control and Prevention CDC. Hepatitis A, acute 2019 case definition. CDC. https://ndc.services.cdc.gov/case-definitions/hepatitis-a-acute-2019/. Accessed 2025 May 1.

[19] Adams C, Kirby AE, Bias M, Riser A, Wong KK, Mercante JW, et al. Detecting Mpox Cases through wastewater surveillance - United States, August 2022-May 2023. MMWR Morb Mortal Wkly Rep. 2024;73(2):37–43. doi: 10.15585/mmwr.mm7302a3 38236784 PMC10803092

[20] Tjon GMS, Coutinho RA, van den Hoek A, Esman S, Wijkmans CJ, Hoebe CJPA, et al. High and persistent excretion of hepatitis A virus in immunocompetent patients. J Med Virol. 2006;78(11):1398–405. doi: 10.1002/jmv.20711 16998883

[21] Mao JS, Yu PH, Ding ZS, Chen NL, Huang BZ, Xie RY, et al. Patterns of shedding of hepatitis A virus antigen in feces and of antibody responses in patients with naturally acquired type A hepatitis. J Infect Dis. 1980;142(5):654–9. doi: 10.1093/infdis/142.5.654 6257794

[22] O’Reilly KM, Verity R, Durry E, Asghar H, Sharif S, Zaidi SZ, et al. Population sensitivity of acute flaccid paralysis and environmental surveillance for serotype 1 poliovirus in Pakistan: an observational study. BMC Infect Dis. 2018;18(1):176. doi: 10.1186/s12879-018-3070-4 29653509 PMC5899327

[23] Masri NZ, Card KG, Caws EA, Babcock A, Powell R, Lowe CJ, et al. Testing specificity and sensitivity of wastewater-based epidemiology for detecting SARS-CoV-2 in four communities on Vancouver Island, Canada. Environ Adv. 2022;9:100310. doi: 10.1016/j.envadv.2022.100310 36321068 PMC9613784

[24] Hewitt J, Trowsdale S, Armstrong BA, Chapman JR, Carter KM, Croucher DM, et al. Sensitivity of wastewater-based epidemiology for detection of SARS-CoV-2 RNA in a low prevalence setting. Water Research. 2022;211:118032. doi: 10.1016/j.watres.2021.118032 35042077 PMC8720482

[25] Hata A, Honda R. Potential sensitivity of wastewater monitoring for SARS-CoV-2: Comparison with norovirus cases. Environ Sci Technol. 2020;54(11):6451–2. doi: 10.1021/acs.est.0c02271 32421334

[26] Thorndike AL, Yetman HE, Thorndike AN, Jeffrys M, Rowe M. Unmet health needs and barriers to health care among people experiencing homelessness in San Francisco’s Mission District: A qualitative study. BMC Public Health. 2022;22(1):1071. doi: 10.1186/s12889-022-13499-w 35637496 PMC9150384

[27] Trudel-Ferland M, Jubinville E, Jean J. Persistence of Hepatitis A virus rna in water, on non-porous surfaces, and on blueberries. Front Microbiol. 2021;12:618352. doi: 10.3389/fmicb.2021.618352 33613487 PMC7890088

