# Supplementary figures and images for: Assessing the sensitivity and predictive value of wastewater in detection of Hepatitis A cases in San Diego County
Source: PLoS One. 2026 Feb 18;21(2):e0342229. doi: 10.1371/journal.pone.0342229 (PMC12915944; doi:10.1371/journal.pone.0342229)

Supplementary Figure 1: HAV Cases and Association to Point Loma Catchment

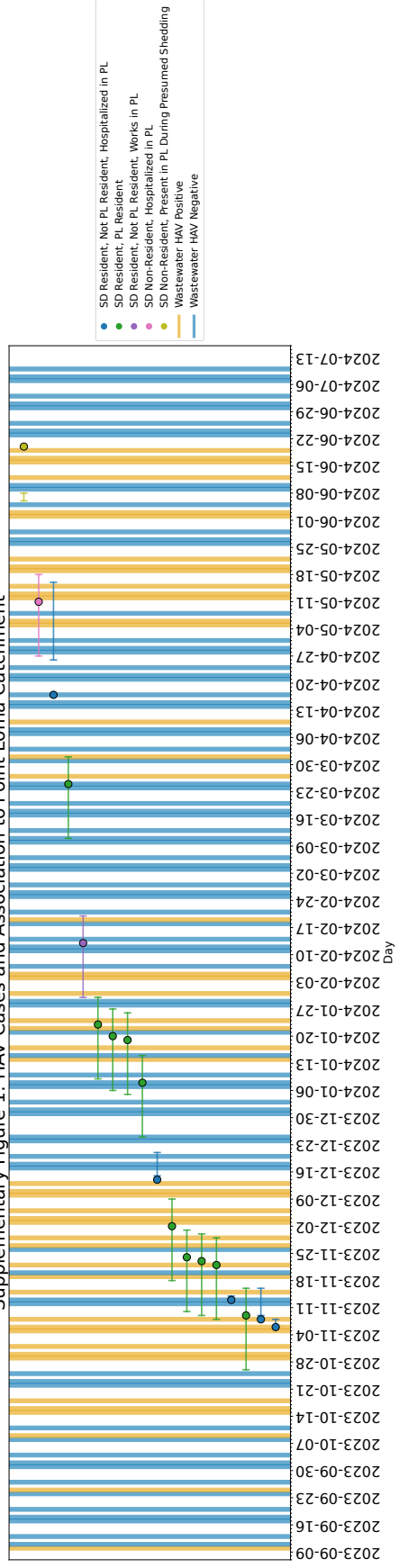

Supplement: S1 Fig — A total of 6 cases were added that worked, were hospitalized or were present during their presumed period of illness. The episode dates and shedding periods of these cases, along with the observed wastewater signal are shown. When these cases are included, there is a higher number of true positives and false negatives. This indicates that the additional cases could be responsible for some wastewater signals, but not all their shedding potentially occurs in the PL catchment area. (PDF) [file pone.0342229.s001.pdf]
