# Supplementary material for: Assessing the sensitivity and predictive value of wastewater in detection of Hepatitis A cases in San Diego County
Source: PLoS One. 2026 Feb 18;21(2):e0342229. doi: 10.1371/journal.pone.0342229 (PMC12915944; doi:10.1371/journal.pone.0342229)
Supplement: S1 Table — (DOCX) [file pone.0342229.s002.docx]

**S1 Table. Sensitivity in Percent as Number of Individuals Shedding Increases for All Wastewater Classification Methods.**

| **Wastewater Signal Classification Strategy** | **Number of Individuals Shedding** | | | |
| --- | --- | --- | --- | --- |
|  | **≥ 1** | **≥ 2** | **≥ 3** | **≥ 4** |
| Observed wastewater signal | 48.1 (34.5, 61.7) | 52.6 (30.1, 75.1) | 50 (26.9, 73.1) | 50 (21.7, 78.3) |
| Wastewater signal reclassified if any positive in the week | 71.2 (58.8, 83.5) | 84.2 (67.8, 100)* | 83.3 (66.1, 100)* | 91.7 (76.1, 100)* |
| Wastewater signal reclassified based on 5-sample trimmed centered average (default) | 69.2 (56.7, 81.8) | 84.2 (67.8, 100)* | 83.3 (66.1, 100)* | 91.7 (76.1, 100)* |
| Wastewater signal reclassified based on 3-sample centered average | 73.1 (61.0, 85.1) | 89.5 (75.7, 100)* | 88.9 (74.4, 100)* | 100 (100, 100) |
| Wastewater signal reclassified based on 5-sample centered average | 78.8 (67.7, 89.9) | 94.7 (84.6, 100)* | 94.4 (83.8, 100)* | 100 (100, 100) |
| Wastewater signal reclassified based on 7-sample centered average | 84.6 (74.8, 94.4) | 100 (100, 100) | 100 (100, 100) | 100 (100, 100) |
| Wastewater signal reclassified based on 3-sample rolling average | 67.3 (54.6, 80.1) | 84.2 (67.8, 100)* | 83.3 (66.1, 100)* | 91.7 (76.1, 100)* |
| Wastewater signal reclassified based on 5-sample rolling average | 67.3 (54.6, 80.1) | 84.2 (67.8, 100)* | 83.3 (66.1, 100)* | 91.7 (76.1, 100)* |
| Wastewater signal reclassified based on 7-sample rolling average | 71.2 (58.8, 83.5) | 84.2 (67.8, 100)* | 83.3 (66.1, 100)* | 91.7 (76.1, 100)* |

*****indicates that upper limit of 95% CI was truncated to 100%, CIs calculated using normal approximation
