# Supplementary material for: Assessing the sensitivity and predictive value of wastewater in detection of Hepatitis A cases in San Diego County
Source: PLoS One. 2026 Feb 18;21(2):e0342229. doi: 10.1371/journal.pone.0342229 (PMC12915944; doi:10.1371/journal.pone.0342229)
Supplement: S2 Table — (DOCX) [file pone.0342229.s003.docx]

**S2 Table. Sensitivity in Percent at Varying Detection Thresholds using Observed Wastewater Signal.**

| **Detection Threshold** | **Sensitivity % (95% CI)** | **PPV % (95% CI)** | **NPV % (95% CI)** |
| --- | --- | --- | --- |
| 1000 cp/g (baseline) | 48.1 (34.5, 61.7) | 52.1 (38.0, 66.2) | 67.5 (57.4, 77.5) |
| 5000 cp/g | 46.2 (32.6, 59.7) | 52.2 (37.7, 66.6) | 67.1 (57.1, 77.1) |
| 7500 cp/g | 38.5 (25.2, 51.7) | 54.1 (38.0, 70.1) | 66.0 (56.4, 75.5) |
| 10000 cp/g | 36.5 (23.5, 49.6) | 59.4 (42.4, 76.4) | 66.7 (57.4, 76.0) |

95% CI calculated using normal approximation.
